# Significant Expansion of Real-Time PCR Multiplexing with Traditional Chemistries using Amplitude Modulation

**DOI:** 10.1038/s41598-018-37732-y

**Published:** 2019-01-31

**Authors:** Aditya Rajagopal, Dominic Yurk, Claudia Shin, Karen Menge, Lucien Jacky, Scott Fraser, Thomas A. Tombrello, Gregory J. Tsongalis

**Affiliations:** 1 Department of Physics, Mathematics, and Astronomy, California Institute of Technology, Pasadena, CA 91125 USA; 2 ChromaCode, Inc., Carlsbad, CA 92008 USA; 3 Dornslife College of Letters, Arts, and Sciences, University of Southern California, Los Angeles, CA 90089 USA; 4 Department of Pathology and Laboratory Medicine, Geisel School of Medicine, Dartmouth Hitchcock Medical Center, Lebanon, NH 03756 USA

## Abstract

The real time polymerase chain reaction (rtPCR) is an essential method for detecting nucleic acids that has a wide range of clinical and research applications. Current multiplexed rtPCR is capable of detecting four to six nucleic acid targets in a single sample. However, advances in clinical medicine are driving the need to measure many more targets at once. We demonstrate a novel method which significantly increases the multiplexing capability of any existing rtPCR instrument without new hardware, software, or chemistry. The technique works by varying the relative TaqMan probe concentrations amongst targets that are measured in a single fluorometric channel. Our fluorescent amplitude modulation method generates a unique rtPCR signature for every combination of targets present in a reaction. We demonstrate this technique by measuring nine different targets across three color channels with TaqMan reporting probes, yielding a detection accuracy of 98.9% across all combinations of targets. In principle this method could be extended to measure 6 or more targets per color channel across any number of color channels without loss in specificity.

## Introduction

The polymerase chain reaction (PCR) is the most widely implemented *in vitro* method for multiplying DNA^1^. The facility, sensitivity, specificity, and reproducibility of PCR have led to its wide use in an array of applications including monitoring of gene expression, detection of somatic mutations, and quantitative measurement of infectious agents such as viruses, bacteria, and parasites^2,3^. A variant of this method, real-time PCR (rtPCR), monitors the progression of DNA amplification by measuring the signal of fluorescent molecules (chromaphores) activated during the PCR process^4^. Modern rtPCR instrumentation and chromaphore chemistries have evolved to allow for the simultaneous measurement of multiple DNA amplification reactions in a single reaction vessel. With multiplexed rtPCR, targets are either discriminated by the PCR product length using melt curves^5^ or multiple detection temperatures (MuDT)^6^, or by wavelength and sequence specific labels such as molecular beacons^7^ or TaqMan probes^8^.

Advances in understanding of complex biology and clinical medicine are driving the need to measure many more DNA sequences in a single sample^9,10^. Sequence specific labeling methods, such as TaqMan rtPCR, have been limited to measuring a single target per reporting chromophore, or four to six DNA targets per reaction^11^. To overcome this limitation, a number of alternate approaches have been attempted which suffer from various drawbacks. For example, some methods introduce new instrumentation which partitions and separates samples, either into many smaller droplets^11–13^ or on to spatially distinct reaction sites^14^. Other methods sacrifice specificity by pooling measurements of multiple genes into a single fluorescent measurement^6,15^. Some approaches are able to discriminate multiple genes in one channel by altering the PCR temperature protocol and exploiting differences in oligo melt temperatures. However, this method is currently limited to distinguishing 2 genes per channel^16^.

To address this gap in rtPCR capability we demonstrate a highly reproducible, maximally-information dense technique for multiplexing rtPCR reactions by adjusting primer and probe levels. This technique improves upon prior work^17^ by improving signal processing techniques, expanding to measure targets across multiple color channels, and demonstrating viability on multiple different rtPCR platforms. The advantage of this method is that it is run on existing rtPCR hardware without any new software, protocols, design processes, or chemistries. This 3x increase in bandwidth without the need for new instrumentation or processes greatly improves efficiency in research and diagnostic labs. Furthermore, the coding technique used in the method is extensible to an arbitrary number of targets per channel, potentially allowing this bandwidth gain to increase to 6x or higher.

## Results and Discussion

### Engineering PCR Endpoint Signal Intensity

**Figure 1:**
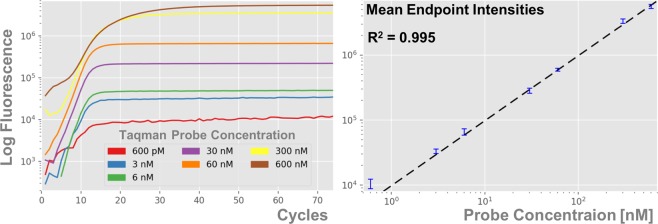
Fluorescent Intensity Scales Linearly with Probe Concentration. End-point fluorescence intensity for each target is set by its TaqMan probe concentration. (**a**) Sample PCR curves detecting the presence of an Influenza B target with different concentrations of TaqMan reporting probe. (**b**) Proportional (*y* = *bx*) regression of endpoint intensity levels generated by various probe concentrations. Twelve replicates were performed at each probe concentration, which were used to generate the means and 1-*σ* error bars shown. The high *R*^2^ value of 0.995 indicates that this is a very strong proportional relationship.

To distinguish multiple targets in a single fluorometric channel we implement a scheme in which each target is associated with a different concentration of fluorescent TaqMan probe. This method of analyzing PCR intensity curves and determining which targets are present in a sample relies on the assumption that measured fluorescence scales linearly with probe concentration. To test this assumption we conducted seven experiments of 12 replicates each amplifying influenza B (Flu-B) with probe concentrations ranging from 600 pM to 600 nM. As shown in Fig. 1 this produced a very good proportional relation between intensity and probe concentration with *R*^2^ = 0.995, *χ*^2^ = 2.00. ${\chi }_{0.95}^{2}=3.84$ for this one d.o.f. test, so our lower *χ*^2^ indicates that our data is a good match to a proportional fit. The only point that deviates significantly from this fit is the 600 fM point, which has higher than predicted intensity. Since the probe is present in such a small concentration this is likely due to background noise, as evidenced by the variation in its fluorescence curve in Fig. 1a. Removing this data point and repeating the fit yields a *χ*^2^ of 1.03, indicating an excellent proportional fit. All of the probe concentrations used in later experiments were well above 600 pM, in the regime where we can rely on a linear relationship between probe concentration and fluorescent intensity.

### Proof of Concept with Spiked Nasopharyngeal Samples

**Figure 2:**
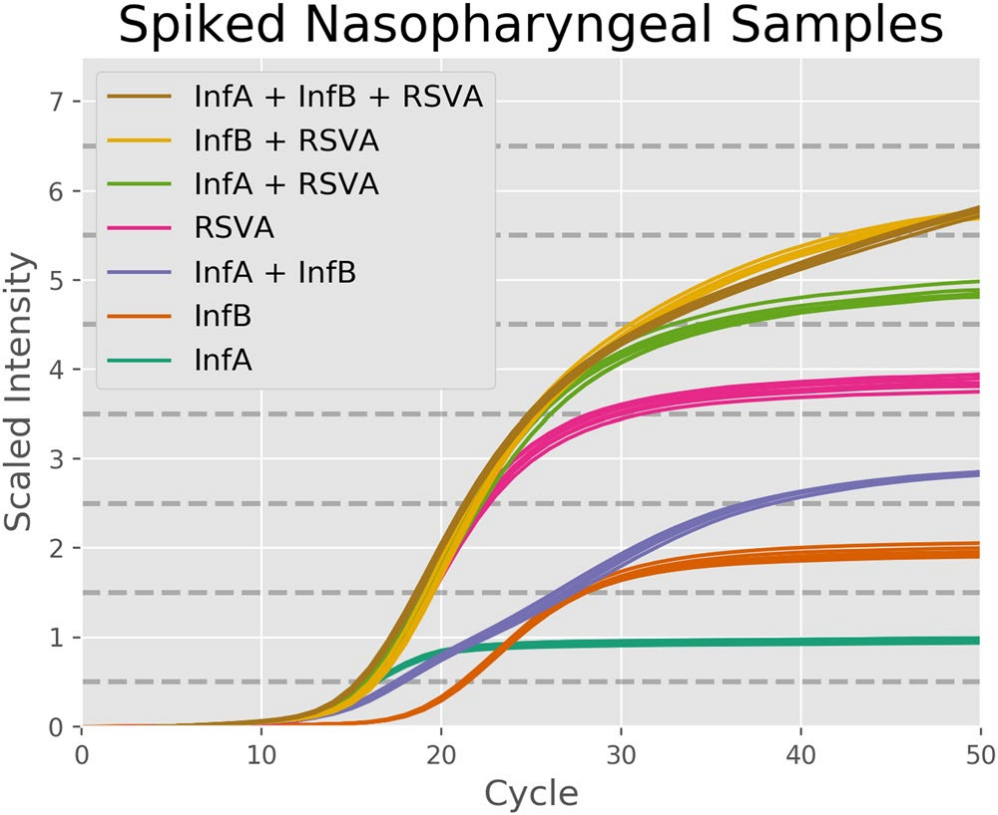
Results from Spiked Nasopharyngeal Samples. This graph shows the results of 42 different experiments using our binary coding method on spiked nasopharyngeal samples, with 6 technical replicates at each target combination. The probes were designed to emit in the FAM channel, and the resultant signals were normalized based off of the ROX channel. All combinations of up to two targets were consistently identified correctly with 100% accuracy. In the samples with all three targets amplification was not completed within 50 cycles, resulting in false negatives for Influenza A.

To demonstrate the applicability of our information dense coding method in a clinical scenario we used it to analyze spiked nasopharyngeal samples. We designed an assay consisting of three targets measured simultaneously in a single rtPCR color channel: influenza A (IA – GenBank Accession: M23976.1; see Table 1), influenza B (IB – GenBank Accession: AB036876; see Table 2), and respiratory syncytial virus A (RA – GenBank Accession: |JX627336.1|:5726-7450; see Table 3). The coding method in this assay leverages the fact that fluorescent intensity scales linearly with digested probe concentration. We scale the probe concentration for each target in powers of 2: 6.25 nM for IA, 12.5 nM for IB, and 25 nM for RA. This results in every possible combination of targets having a unique total digested probe concentration, and therefore a unique fluorescent intensity level. Using this one-to-one mapping, we can extract a fluorescent intensity from a PCR curve corresponding to an unknown sample and use it to determine the exact set of targets present. The results are shown below in Fig. 2. In this experiment we were able to consistently identify all possible combinations of up to two different targets in a single color channel with 100% accuracy across 36 samples. This identification was performed using the endpoint matching method described below. When all three targets were present at once the replicates were still tightly grouped, but they were not able to complete their amplification within 50 cycles. The result was a false negative for IA in all of these samples. This problem could be resolved by running the PCR for more cycles or, preferably, by improving the chemistry system to increase PCR efficiency. The latter approach is demonstrated in the following section.

**Table 1:** Influenza A sequence information.

Sequence Information	Influenza A
Source	GenBank Accession # M23976.1 Influenza A virus (A/Ann Arbor/6/1960(H2N2)) nucleoprotein gene, complete cds
85Mer Template	5′ GTA GGG ATA GAC CCT TTC AAA CTG CTT CAA AAC AGC CAA GTA TAC AGC CTA ATC AGA CCG AAT GAG AAT CCA GCA CAC AAG AGT C 3′
FWD Primer	5′ GTA GGG ATA GAC CCT TTC AAA CTG 3′
RWD Primer	5′ GAC TCT TGT GTG CTG GAT TCT C 3′
TaqMan Probe	5′/56FAM/AG CCA AGT ATA CAG CCT AAT CAG ACC GA/3BHQ_1/3′

**Table 2:** Influenza type B sequence information.

Sequence Information	Influenza B
Source	GenBank Accession # AB036876 Influenza B virus (B/Nagoya/20/99) NP gene for nucleoprotein
81Mer Template	5′ GTG CTT CCC ATA AGC ATT TAC GCC AAA ATA CCT CAA CTA GGG TTC AAC GTT GAA GAG TAC TCT ATG GTT GGG TAT GAA GCC 3′
FWD Primer	5′ GTG CTT CCC ATA AGC ATT TAC G 3′
RWD Primer	5′ GGC TTC ATA CCC AAC CAT AGA G 3′
TaqMan Probe	5′/56FAM/CC TCA ACT AGG GTT CAA CGT TGA AGA GT/3BHQ_1/3′

**Table 3:** Respiratory syncytial virus type A sequence information.

Sequence Information	Respiratory syncytial virus type A
Source	GenBank Accession #|JX627336.1|:5726-7450 Human respiratory syncytial virus strain RSVA/GN435/11, complete genome
81Mer Template	5′ GTT GGA AAC TAC ACA CAT CTC CTC TAT GTA CAA CCA ACA CAA AGG AAG GAT CCA ACA TCT GCT TAA CAA GAA CCG ACA GAG GAT G 3′
FWD Primer	5′ GTT GGA AAC TAC ACA CAT CTC CTC 3′
RWD Primer	5′ CAT CCT CTG TCG GTT CTT GTT AAG 3′
TaqMan Probe	5′/56FAM/CC AAC ACA AAG GAA GGA TCC AAC ATC TG/3BHQ_1/3′

### Expansion to Multiple Color Channels

**Figure 3:**
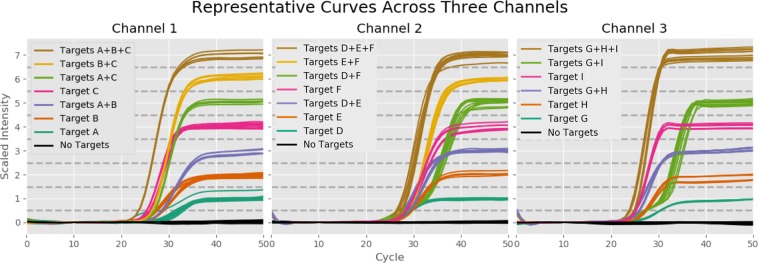
Representative 9-Plex Curves. These graphs show a representative set of curves from our 9-Plex test across all three color channels. In each of these curves all targets are present at 10^4^ copies (except for the A + C curves in channels 2 and 3, in which target C was present at 100 copies). The different target combinations tested produce consistent and distinct PCR curves across all color channels, allowing us to identify the targets present in each sample with high accuracy.

In order to build off of the proof of concept described above, we designed an assay capable of detecting nine distinct targets across three different color channels, as laid out in Table 4. This expansion in the number of targets being tested was accompanied by improvements to the chemistry system, including adoption of a new polymerase master mix and a significant increase of all primer and probe concentrations. We tested our target detection methods on 1030 synthetic samples across 14 plates. These samples included dilution series of all nine individual targets from 10^5^ down to 6.25 copies/sample, as well as nine different 2-target combinations and three different 3-target combinations. Figure 3 shows a representative set of PCR curves generated by these samples. This illustrates that different target combinations are producing consistent and distinct signals across all three color channels. In particular, even the samples with three different targets present are completing their amplification by cycle 40, eliminating the false negative problem seen in the earlier data set.

**Table 4:** 9-Plex Target Layout.

Channel	Target A (Level 1)	Target B (Level 2)	Target C (Level 4)
Channel 1 (FAM)	Influenza B	Respiratory Syncytial Virus A	Respiratory Syncytial Virus B
Channel 2 (ATTO532)	Influenza A	Influenza A Subtype H3	Influenza A Subtype H1
Channel 3 (ATTO647N)	Parainfluenza 2	Parainfluenza 3	Parainfluenza 1

**Figure 4:**
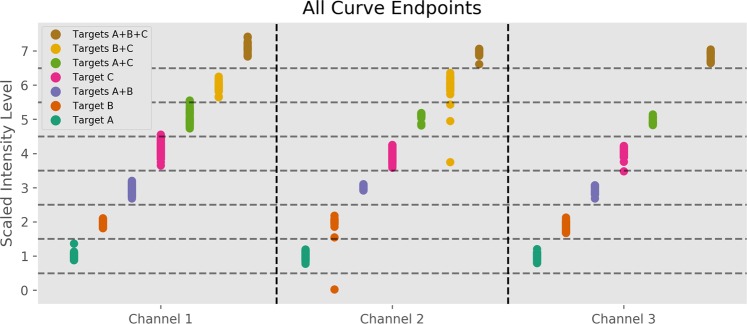
Classifying Sample Curves Using PCR Endpoint. This graph shows the endpoint of every one of our 796 PCR curves across all three color channels, excluding negative detections. The horizontal dashed lines represent the boundaries of the ‘bins’ used to match each curve to a level. For example, any sample with scaled endpoint intensity landing between 2.5 and 3.5 is identified as being in level 3, indicating that it contains targets A and B. The fact that all A + B samples in all color channels landed between these boundaries indicates that all A + B samples were classified correctly.

The dilution series were used to establish a limit of detection (LOD) for each individual target, which was defined for these purposes as the minimum concentration at which all samples tested amplified to the proper level. The measured LODs for the nine targets ranged from 10 to 100 copies/sample. All samples with target concentrations below LOD were removed from the data set. Of the remaining 796 samples, 787 (98.9%) were matched to the correct set of targets using endpoint matching (as illustrated in Fig. 4). These samples covered a concentration range of at least 10^2^ to 10^5^ copies/sample for each individual target. Furthermore, relative target concentrations were varied over a wide range in the two-target samples, up to a ratio of 1000:1. This indicates that our method is highly robust across a wide range of target concentrations.

**Figure 5:**
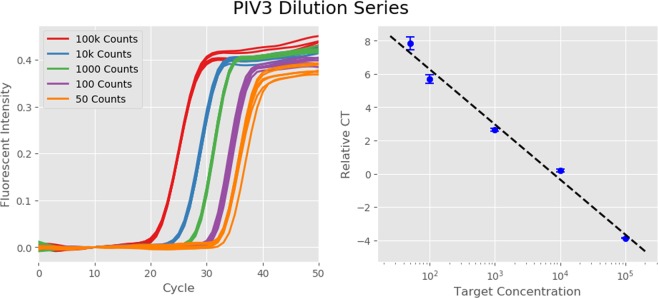
Dilution Series of a Single Target. Panel (a) shows the PCR curves from a dilution series of Parainfluenza Virus 3 from 10^5^ down to 50 copies/sample, with 6 replicates at each concentration. The tight grouping among replicate curves indicates that our assay should produce consistent CT values from run to run. Panel (b) confirms this by showing the average CTs of the replicates at each concentration relative to a standard at 10^4^ copies, along with 1-*σ* error bars. Not only do the small error bars indicate tight grouping, but the points are well matched to a linear fit of CT vs. log(Concentration). This indicates that quantitation could be potentially viable when at most one target amplifies per color channel.

We have shown that our method can consistently determine the presence or absence of many targets in a sample. However, in some scenarios it is also necessary to determine the approximate concentration of each target which is present. This is traditionally done by measuring the cycle at which a PCR curve attains its maximum second derivative and comparing the resultant cycle threshold (CT) to that of a standard with known concentration. In order for this method to produce consistent results, different technical replicates of a sample with the same concentration must produce consistent CTs. A dilution series and set of relative CTs for one of the targets in our 9-plex is shown in Fig. 5. When compared to a standard at 10^4^ copies, this dilution series produces a very good linear fit of CT vs. log(Concentration). This indicates that quantitation is readily possible using our method if at most one target amplifies in a given channel. However, searching for the maximum of the second derivative will not be sufficient for quantitation of multiple different targets amplifying in the same channel. In principle, curves produced by multiple target amplification could be broken down into their individual components to extract a separate CT for each target, but that is beyond the scope of this study.

### Extension to Other PCR Platforms

All of the data shown above were collected on Applied Biosciences (ABI) PCR systems from ThermoFischer Scientific. In order to demonstrate that our technique is viable across multiple PCR platforms, we also conducted a test of our 9-Plex assay on the Roche LightCycler 480 system. This test consisted of 172 samples across two plates and covered a dilution series of every individual target from 10^5^ down to 100 copies as well as five different two-target combinations and one three-target combination, with each condition repeated across at least three wells. The chemistry system, PCR cycling parameters, normalization, and analysis methods used were the exact same as the ones used on the ABI 9-Plex tests above. Two of the samples were thrown out due to clear signs of contamination. Of the remaining 170 samples, 168 (98.8%) were matched to the correct set of targets. In particular, 26 of the 27 samples with a single target at a concentration of 100 counts were called correctly, and all three replicates of the three-target sample were called correctly. This high level of accuracy indicates that our method can easily be transferred between different PCR platforms without the need for chemistry reformulation or any tuning of the cycling conditions or analysis parameters.

## Methods

### Materials and PCR Parameters

For the nasopharyngeal POC, the samples were residual anonymized and de-identified clinical samples for which our lab had both the use-rights and access. As such, IRB approvals were not needed for this study. Primers and TaqMan reporting probes for all targets (P3, IB, and RA) were designed using Integrated DNA Technology (IA, USA) OligoAnalyzer Tool. Each of the TaqMan probes was designed to have 6-FAM reporting chemistry. For all PCR reactions, each target sequence that was present had a concentration of 31 pM. P3 had forward and reverse primers at concentrations of 31 nM and fluorescent probes at a concentration of 6.25 nM. IB had primer and probe concentrations twice that of P3 (62 nM and 12.5 nM), and RA had primer and probe concentrations four times that of P3 (124 nM and 25 nM). All PCR reactions where performed in an ABI 7500 thermocycler with 10 minutes of initial denaturation at 95 °C followed by 50 cycles with 15 s at 95 °C, 30 s at 58 °C, and 60 s at 75 °C. All signal acquisition was conducted at 58 °C.

For the 9-Plex assay, all primers and probes were also designed using the OligoAnalyzer Tool. The TaqMan probes were designed to report in either FAM, ATTO532, or ATTO647 depending on the target. Primer concentrations for all targets were set to 2 *μ*M. Probe concentrations in each channel were set to 31.25 nM for target A, 62.5 nM for target B, and 125 nM for target C. All PCR reactions where performed in either an ABI 7500 or ABI QS7 thermocycler with one minute of initial denaturation at 95 °C followed by 50 cycles with 15 s at 95 °C and 120 s at 60 °C. All signal acquisition was conducted at 60 °C.

### Amplitude Modulation Coding Method

**Figure 6:**
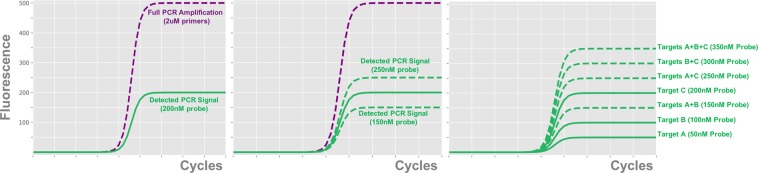
Illustration of Amplitude Modulation Coding. (**a**) Traditional PCR amplification. The low ratio of probe to primer concentration causes all probe to be digested over the course of amplification, resulting in an fluorescent signal proportional to the initial probe concentration. (**b**) The intensity of the output signal from PCR can be modulated by altering the initial concentration of probe present in the reaction. (**c**) By adding probes for multiple different targets to the reaction at increasing concentrations, we can make each combination of targets correspond to a unique total amount of probe. This causes each combination of targets to produce a unique total fluorescent signal at the end of amplification.

Figure 6 illustrates the chemical basis of our amplitude modulation coding method. Panel (a) shows a traditional single-target PCR chemistry. In this system the primer is at a significantly higher concentration than the probe, so as PCR proceeds all probe in the sample will be digested and generate signal. Panel (b) illustrates the concept that the amplitude of the total signal produced by a sample varies linearly with the amount of probe which is present. This allows us to set the amplitude produced by a given target by setting the concentration of the probe associated with that target at the proper level.

In our coding scheme we scale up probe concentration from target to target by powers of two. The result of this is demonstrated in panel (c), which shows that each possible combination of targets corresponds to a distinct total amount of digested probe. This coding scheme is maximally information dense, and is at the Shannon information limit^18^. In order to simplify description of the signal amplitudes produced by each target combination, we refer to them by “levels”; the individual targets A, B and C are said to amplify to levels 1, 2, and 4, the combination A + B is said to amplify to level 3, and a no-target sample is said to be at level 0. Since each target combination corresponds to a unique level, the problem of identifying the set of targets present in a PCR well is reduced to mapping the output curve of the sample to a level from 0 to 7. The remaining sections are devoted to describing our mapping method. While we demonstrate the method in this study with three targets per color channel, this technique could easily be extended to any number of targets per channel provided there is low enough noise.

### Target Detection with Endpoint Matching

In order to determine which targets are present in a given sample we need to assign its generated fluorescence curve a level from 0 to 7. The first step is to normalize the signal produced by the PCR instrument to correct for well-to-well variations in optical sensitivity, pipetting volume, and other anomalous effects. In the nasopharyngeal POC experiments it was found that the most effective normalization method was to divide each cycle by the amplitude in the ROX channel, which was meant to act as a passive reference. This type of passive reference normalization is commonly applied by commercial software from rtPCR machine vendors. However, in the 9-Plex experiments this ROX normalization was found to be ineffective, possibly due to the large degree of crosstalk between the ATTO532 and ROX channels. In order to avoid this problem we instead implemented a “self-normalization” technique, whereby the PCR curve in each channel was divided by the median of its raw amplitude measurements in that channel from cycles 6 to 9. Since none of our curves showed any significant amplification before cycle 15, this 6–9 window served as an effective measure of the background level of fluorescence in each channel. This background level performs a similar function to the passive background level from the ROX channel in the nasopharyngeal POC test, allowing us to eliminate much of the well-to-well variation between replicates.

Once our curves were normalized we needed to assign them to the proper level. This was done using standards. In the 9-Plex assay, 9 wells on each plate were designated as “calibrators”, and each one contained exactly one of the nine targets present at a concentration of 10^4^ copies. There was one additional calibrator well with no targets which served as a negative control. This provided us with a reference of what amplitude to expect from each target, thereby allowing us to assign an amplitude to levels 0, 1, 2, and 4 in each channel based on endpoint intensities. For example, level 4 in channel 1 is mapped to the endpoint intensity of the target C standard in channel 1. Once these single-target levels were determined, we constructed multi-target levels by adding them together and subtracting out the double-counted background. For example, the amplitude of level 3 was set to (Level 1) + (Level 2) − (Level 0).

Once all levels across all channels were calculated from standards we could match a PCR curve from an unknown sample to the proper level. The amplitude of each unknown curve was measured at its endpoint, and the curve was assigned the level which had an amplitude most closely matching its own endpoint amplitude. For example, in the figures above the amplitudes of the standards have been scaled such that level 1 falls at 1.0, level 2 falls at 2.0, and so on. This means that any unknown sample curve with an endpoint falling between 1.5 and 2.5 was classified as a level 2, and any curve falling between 5.5 and 6.5 was classified as a level 6. Finally, once a sample was assigned an independent level in each of the color channels being used we mapped those levels back to a unique set of targets which were present in the sample.

## Conclusion

In conclusion, we have demonstrated a method of increasing the multiplexing capability of existing rtPCR instruments without the need for new hardware, software, or chemistry. This method improves significantly over existing high-bandwidth PCR techniques by maintaining both high target detection accuracy and the ability to detect multiple targets simultaneously without significantly altering routine PCR workflow. In theory our combinatorial coding and normalization techniques can be extended to any number of targets per channel. The linearity relation between probe concentration and fluorescent intensity extends over at least 2 orders of magnitude, which would allow the encoding of at least 6 different targets. However, accurately identifying *n* targets per channel requires the ability to distinguish between 2^*n*^ different intensity levels. Such an exponential scaling in the required precision mandates more sophisticated techniques to remove noise from the PCR signal. This noise reduction could come from improvements in instrumentation, chemistry design, or data processing and analysis techniques. As these factors improve, we anticipate that our method would readily enable standard rtPCR instruments to multiplex 12 to 24 targets per reaction across four channels.
